# Neighborhood built environment, psychosocial stressors, and telomere length of birth parents and their newborns from San Francisco, California

**DOI:** 10.1038/s41370-025-00797-9

**Published:** 2025-08-08

**Authors:** Lara J. Cushing, Hasibe Caballero-Gomez, Stephanie M. Eick, Ana C. Pelegrini Guimaraes, Nicholas J. Depsky, Erin DeMicco, Jue Lin, Tracey J. Woodruff, Rachel Morello-Frosch

**Affiliations:** 1 Department of Environmental Health Sciences, Fielding School of Public Health, University of California, Los Angeles, CA USA; 2 Gangarosa Department of Environmental Health, Rollins School of Public Health, Emory University, Atlanta, GA USA; 3 Department of Geography & Environment, San Francisco State University, San Francisco, CA USA; 4 Energy and Resources Group, University of California, Berkeley, Berkeley, CA USA; 5 Program on Reproductive Health and the Environment, Department of Obstetrics, Gynecology and Reproductive Sciences, University of California, San Francisco, CA USA; 6 Department of Biochemistry and Biophysics, University of California San Francisco School of Medicine, San Francisco, CA USA; 7 Department of Environmental Science, Policy and Management and School of Public Health, University of California, Berkeley, Berkeley, CA USA

**Keywords:** Cumulative impacts, Environmental justice, Geospatial analyses, Maternal and fetal exposure/health, Mixtures, Non-chemical stressors, Noise pollution, Green space

## Abstract

**Background:**

Shorter telomere length is a biomarker of cellular aging influenced in early life. Exposure to environmental hazards and psychosocial stressors disproportionately impact socially marginalized populations and have been linked with shorter telomeres.

**Objective:**

To estimate joint associations between residential neighborhood greenness, traffic, noise, and perceived neighborhood quality, psychosocial stress and depression on telomere length of birth parents and their newborns.

**Methods:**

Telomere length (T/S ratio) was measured in leukocytes from 354 2nd trimester parental and 488 umbilical cord blood samples collected at delivery from the Chemicals in Our Bodies cohort in San Francisco, California. Normalized difference vegetation index (NDVI), traffic volume, and noise were estimated based on residential address. Perceptions of neighborhood quality, psychosocial stress, and depression were collected via questionnaire. We used quantile g-computation to assess joint associations between all exposures and newborn and parental T/S in separate models controlling for parental age, race and ethnicity, education, pre-pregnancy body mass index, and gestational age (cord T/S only). We used interaction terms to assess effect measure modification by nativity, race and ethnicity, and educational attainment.

**Results:**

Parental and newborn T/S were not correlated with individual measures of built environment or psychosocial stressors (rho from −0.08 to 0.08). A simultaneous one quartile increase in all adverse exposures was associated with a decrease in newborn T/S (mean difference [95% CI] = −0.03 [−0.08, 0.01]) that was stronger when restricting to paired parental-newborn samples and controlling for parental T/S (−0.08 [−0.15, −0.01]). Interaction analysis revealed stronger associations among immigrant (−0.08 [−0.16, 0.00]) vs. US-born (−0.02 [−0.07, 0.04]) and college-educated (−0.07 [−0.12, −0.02]) vs. non-college educated (0.03 [−0.07, 0.12]) participants. We saw no association with parental telomere length.

**Significance:**

Results suggest exposure to adverse neighborhood built environments and individual-level psychosocial stressors during pregnancy is associated with reductions in telomere length among newborns.

**Impact:**

Telomere length at birth predicts relative telomere length in adulthood, suggesting much of the link between telomere length and longevity is established early in life. While neighborhood environments have been linked with shorter telomeres in adulthood, few prior studies have assessed newborn telomere length or joint associations with psychosocial stressors. In a diverse birth cohort, we show that the mixture of neighborhood lack of greenness, traffic, and noise, coupled with individual-level poor perceptions of neighborhood quality, stress, and depression is associated with decreased telomere length among newborns, with slightly stronger effects among immigrants and college-educated birth parents.

## Introduction

Shorter telomeres are a biomarker of cellular aging associated with increased risk of multiple aging-related diseases and death [1, 2]. Telomeres are non-coding DNA regions that protect the ends of chromosomes and naturally shorten over time with cell replication. However, telomeres can also shorten due to factors like inflammation and oxidative stress [3, 4]. Chronic stress, early life adversity, and environmental factors such as air pollution have all been associated with shorter telomeres and/or telomere attrition in adulthood [5, 6].

Telomere length at birth is highly variable and predictive of relative telomere length as an adult [7]. When comparing leukocyte telomere length across individuals in adulthood, relative rankings change very little over time [8]. This suggests that much of the link between telomere length and longevity is established early in life. Lower rates of telomere attrition have also been observed among the children of parents with longer telomeres [7]. These factors underscore the importance of identifying modifiable determinants of telomere length for pregnant people and their infants at birth [9].

The neighborhood environment strongly influences a person’s exposure to environmental hazards and social stressors that have been linked to telomere length. The environmental justice literature documents the confluence of and interaction between environmental and social stressors to health in ethno-racially and socioeconomically marginalized populations, contributing to health disparities that could be reflected in differences in telomere length [10]. Prior studies have associated residential particulate air pollution and proximity to major roads with shorter telomere length in newborns [11, 12], potentially through mechanisms involving chronic inflammation, which promotes cell turnover and oxidative stress [3]. Traffic-related air pollution is known to impact both inflammation and cellular stress [13]. Residential noise exposure has been associated with maternal anxiety and depression [14, 15], and psychosocial stressors have been shown to influence telomere length [5, 16, 17]. Meanwhile neighborhood greenspace has been associated with slower telomere attrition in children [18]. Longer telomeres in adults [19–24] and slower telomere attrition in children [25] have also been linked with neighborhood satisfaction and favorable perceptions of neighborhood safety, order, and/or social cohesion, although findings are not consistent across all studies or populations, and we are aware of only one prior study from our group that has assessed perceptions of neighborhood quality in relation to telomere length in newborns [26].

We used data from a San Francisco, California birth cohort to better understand the influence of the built environment and psychosocial stressors on telomere length during pregnancy and in newborns at delivery. We employed mixture methods to examine the joint association between residential greenspace, traffic, noise, and individual-level measures of psychosocial stressors including perceived neighborhood quality on telomere length in birth parents and their newborns. We hypothesized that residence in neighborhoods with less vegetation, more traffic or more noise, as well as poorer perceived neighborhood quality, perceived stress, and depression would all be associated with shorter telomere length among birth parents and newborns. Since race, ethnicity, and socioeconomic status are often correlated with residential proximity to environmental hazards and a paucity of urban access to greenspace [27, 28], we hypothesized that associations in our study would be stronger among immigrants, ethno-racially minoritized parents, and parents with lower levels of educational attainment.

## Subjects and methods

### Study population

The Chemicals in Our Bodies (CIOB) cohort is an ongoing prospective birth cohort study of the cumulative effects of environmental chemicals and psychosocial stressors on fetal growth and child development. Participants have been recruited since 2014 from three hospitals in San Francisco, CA during their 2^nd^ trimester of pregnancy [29]. Pregnant individuals were eligible if they were 18 years of age or older, spoke English or Spanish, were expecting a singleton birth, and did not have a diagnosed pregnancy complication. The present analysis includes the subset of CIOB participants with a residential address that could be geo-coded and who had umbilical whole blood samples and/or venous whole blood samples collected and analyzed for newborn (*n* = 488) and/or parental (*n* = 354) telomere length, respectively (Supplementary Fig. S1). Parental leukocytes were obtained from 2^nd^ trimester blood samples while newborn samples were collected at delivery.

### Built environment measures

We estimated characteristics of the neighborhood built environment based on the parent’s geocoded residential address reported at birth. We utilized the Terra Moderate Resolution Imaging Spectroradiometer (MODIS) normalized difference vegetation index (NDVI) product to derive as an estimate of neighborhood greenness. NDVI is a remotely sensed measure of primary plant productivity equal to the ratio of the difference between near-infrared and visible light to the sum of these two measures. It ranges from −1.0 to 1.0 with larger values indicating higher levels of vegetation and photosynthetic activity. The MODIS product provides 16-day composite NDVI images at 250 m × 250 m resolution using high-quality pixels from daily, atmosphere-corrected observations [30]. We chose an image from March 5–20, 2016 to characterize residential greenness for CIOB participants because it corresponds roughly with the midpoint of pregnancies included in this study, corresponds to the end of the wet season when vegetation is at its peak and thus easiest to measure, and is not overly affected by building shadows due to sun inclination which are highest in winter. The mean NDVI within a 300 m circular buffer distance of the study participant’s home was calculated using Zonal Statistics in ArcGIS (ESRI, Redlands, CA). The 300 m distance was selected based on two recent meta-analyses suggesting the associations between birthweight and NDVI at that distance [31, 32] Negative NDVI values generally indicate water and were suppressed prior to averaging so as not to downwardly bias estimates for participants residing near the coast.

We estimated neighborhood traffic volume (average number of trips per month within 200 m of the home) using data on passenger vehicle and commercial truck trips from StreetLight Data. StreetLight Data metrics are derived from empirical-statistical modeling of space-time data from millions of GPS-enabled mobile devices and have been validated against other more costly and infrequent conventional methods of estimating traffic density including license plate surveys and roadway vehicle counts [33, 34]. We considered trips within a 200 m circular buffer distance from the participant’s residential address in light of prior studies finding most traffic-related pollutant concentrations decay to background levels within a few hundred meters of roadways [35–37]. The traffic metric corresponds to the estimated number of motorized vehicles that passed through, started, or stopped within each buffered zone per month and was generated using a “Zone Activity Analysis” on the StreetLight Data analysis platform. We derived a weighted average across the duration of pregnancy for each participant from these monthly counts. Based on consultation with StreetLight staff, we performed additional calibration of truck trip volumes to improve accuracy using loop counters at two San Francisco locations from the California Department of Transportation public data portal [38]. A calibration factor was derived by comparing data from these two loop counters to StreetLight traffic volume estimates at the same location and then applied to all locations in our analysis. Cohort pregnancies spanned from 2013 to 2019, but we used 2019 monthly traffic data to estimate traffic exposure for all participant because our interest was primarily in spatial (neighborhood) differences in traffic volume which are primarily influenced by the location of major roadways and do not vary significantly year-to-year, and because StreetLight metric accuracy improved over time due to the incorporation of location-based services that became available in 2016, increased sample size, and improved algorithms.

Exposure to noise from transportation was assessed using 24-hour equivalent sound level noise exposure estimates from the National Transportation Noise Map from the Bureau of Transportation Statistics. The map provides A-weighted decibel levels, starting at 45db(A), at 30 × 30 m resolution to better represent human auditory perception, approximating average noise energy due to transportation sources (road and aviation) [39]. Parents were assigned the value of the pixel within which they resided.

### Psychosocial stress and stressors

Information on perceptions of neighborhood quality, perceived stress and depression were obtained via a standardized questionnaire administered by study personnel during a 2nd-trimester prenatal care visit. Neighborhood quality was measured using a previously validated instrument including fifteen questions related to perceptions of collective efficacy, neighborhood safety, neighborhood satisfaction, and physical order [26]. Each question included Likert scale response scored 1 (strongly disagree) to 5 (strongly agree), with positively worded statements reverse coded so that higher scores always corresponded with poorer perceived neighborhood quality. Scores were summed across questions and ranged between 15 and 69. Perceived stress was measured using the 4-item Perceived Stress Scale, with values ranging from 0 to 16 [40], where a higher score corresponds to perceptions of life as more uncontrollable, unpredictable, and overloading. Depression was measured using the 10-item Center for Epidemiologic Studies-Depression [41], a clinical screening tool that measures how often participants experience depression symptoms, with scores ranging from 0 to 30, according to the Diagnostic Statistical Manual-IV.

### Telomere length

Parental venous whole blood samples were collected during a 2^nd^-trimester prenatal visit. Umbilical cord blood was collected at delivery. Average telomere length was measured in genomic DNA extracted from frozen samples at the University of California, San Francisco Blackburn Lab using a quantitate polymerase chain reaction (PCR) assay methods described previously [42]. Average telomere length is defined as the relative ratio of telomere repeat abundance to single-copy gene abundance (T/S ratio). All samples were measured twice with triplicate wells. Lab personnel were blinded to all demographic and clinical data.

### Covariates

Self-reported country of birth, racial and ethnic identity, and educational attainment were collected via the second-trimester questionnaire. Birth parent’s age, gestational age in weeks, infant sex, and pre-pregnancy body mass index were abstracted from participants’ medical records.

### Statistical analysis

We reviewed descriptive statistics for all variables of interest across newborn, parental, and paired parent-newborn observations. We compared values of our exposure and outcome variables between immigrants and US-born participants and across categories of educational attainment and race and ethnicity using the Kruskal–Wallis Rank Sum and Wilcoxon–Mann–Whitney tests because most variables were not normally distributed. We used Spearman’s correlation coefficients to assess the degree of correlation between all variables.

We used quantile g-computation to examine the joint association between our measures of the built environment and psychosocial stressors and the outcome of telomere length. Telomere length of birth parents and their newborns were considered in separate models. We used the qgcomp package in R to estimate the effect of simultaneously increasing all exposures placed in a defined mixture by one quantile on the change in telomere length using a parametric, generalized linear model based implementation of g-computation [43]. Quantile g-computation is a statistical technique used to assess the joint effects of exposure mixtures on an outcome variable. One of its strengths, as compared to other approaches to mixture analysis, is that it allows for exposure-outcome relationships with opposite directions among variables in the mixture. In order to do this, exposures are divided into quantiles based on their distributions and fitted on a regression model where the outcome is modeled as a function of the quantile transformed exposures, adjusting for confounders. The joint effect of exposures is estimated as a weighted sum of the individual exposure contributions, assigning each variable a positive or negative weight equivalent to the proportion of the partial effect in the positive or negative direction, with weights obtained from the regression coefficients, representing each exposure’s relative contribution to the outcome [43]. Weights in each direction (positive and negative) each sum to one and the magnitude of positive weights cannot be compared to the magnitude of negative weights. For ease of comparing relative variable weights, we reverse coded NDVI in the mixture models by subtracting values from the maximum observed value so that higher values of all variables corresponded with conditions we hypothesized were detrimental (less greenspace, more traffic and noise, poorer perceived neighborhood quality, etc.). We controlled for the following covariates based on evidence of associations with telomere length in prior studies: parental age, race and ethnicity, education, pre-pregnancy BMI, and gestational age (for newborn models only). In particular, prior studies show telomeres shorten with age and have observed longer telomeres among individuals with higher BMI, Black race and Latina ethnicity [44–47]. We restricted to participants with complete exposure data for built environmental and psychosocial stressor variables to ensure the quantiles for each exposure would be calculated on the same sample of individuals [43].

We ran additional models with interaction terms for nativity, race and ethnicity, and educational attainment using the qgcompint package to test for effect measure modification and because we observed differences in built environment and psychosocial stressor values in our study population with respect to these factors. When examining effect measure modification by race and ethnicity, parents who identified with an ethnically or racially minoritized population in the U.S. (Asian, Black, Latina, Pacific Islander, Native American, or multi-racial) were grouped and compared to parents who identified as non-Hispanic White due to small sample sizes for some specific racial and ethnic groups (see Table 1). Educational attainment categories were collapsed into yes or no college degree to similarly minimize issues with small cell counts (see Table 1). Given the diversity of the immigrant population in our sample, we additionally examined effect modification by nativity in models stratified by race or ethnicity and educational attainment.

**Table 1:** Characteristics of the study population.

	Newborn (*N* = 488)	Parent (*N* = 354)	Paired (*N* = 222)
Parental age, mean (range) years	34 (16–47)	33 (16–47)	34 (16–47)
Gestational age, mean (range) weeks	39.0 (31.0–42.0)	38.8 (28.0–41.0)	39.0 (31.0–41.0)
Education, *N* (%)			
< High School (HS)	36 (7.4%)	43 (12.1%)	17 (7.7%)
HS Degree or Some College	88 (18.0%)	84 (23.7%)	37 (16.7%)
College Degree	140 (28.7%)	87 (24.6%)	64 (28.8%)
Graduate Degree	211 (43.2%)	120 (33.9%)	95 (42.8%)
Missing	13 (2.7%)	20 (5.6%)	9 (4.1%)
Race and ethnicity, *N* (%)			
White	215 (44.1%)	132 (37.3%)	103 (46.4%)
Latina	130 (26.6%)	139 (39.3%)	61 (27.5%)
Asian	95 (19.5%)	53 (15.0%)	39 (17.6%)
Other/Multi-Racial^a^	21 (4.3%)	15 (4.2%)	9 (4.0%)
Black	20 (4.1%)	10 (2.8%)	6 (2.7%)
Missing	7 (1.4%)	5 (1.4%)	4 (1.8%)
Immigrant, *N* (%)			
Yes	200 (41.0%)	149 (42.1%)	86 (38.7%)
No	277 (56.8%)	177 (50.0%)	127 (57.2%)
Missing	11 (2.3%)	28 (7.9%)	9 (4.1%)
Parental telomere length (T/S), mean (range)	1.0 (0.6–1.8)	1.0 (0.6–1.8)	1.0 (0.6–1.8)
Newborn telomere length (T/S), mean (range)	1.5 (0.8–3.3)	1.4 (0.9–2.1)	1.4 (0.9–2.1)
NDVI within 300 m, mean (range)	0.30 (0.1–0.7)	0.39 (0.1–0.7)	0.31 (0.1–0.7)
Traffic within 200 m, mean (range)	575,569 (15,182–2,615,762)	609,370 (15,182–2,620,886)	583,547 (15,182–2,615,762)
Noise, mean (range) dBA	47.2 (24.8–64.2)	47.4 (24.8–64.2)	47.1 (24.8–64.2)
Neighborhood quality, mean (range)^b^	38.8 (15.0–74.0)	38.7 (15.0–71.0)	38.0 (15.0–61.0)
Perceived stress, mean (range)	5.1 (0.0–13.0)	5.2 (0.0–15.0)	5.1 (0.0–12.0)
Depression, mean (range)	6.7 (0.0–28.0)	6.9 (0.0–26.0)	6.6 (0.0–26.0)

^a^Includes Pacific Islanders, Native Americans, and other groups including multi-racial participants.

^b^Higher values indicate poorer perceived neighborhood quality.

We conducted several sensitivity analyses. First, we added infant sex as a covariate in models of newborn telomere length given a prior study finding an association between sex and telomere length among Latina newborns [48] and prior work in this cohort in which we found telomere length among male infants was more strongly correlated with exposure to exogenous chemicals in utero [42]. Second, we repeated the analysis of newborn telomere length omitting one significant outlier with high T/S ratio to ensure the outlier was not driving any observed associations. Third, we repeated the analysis using only paired parental/newborn samples and controlling for parental T/S in models of newborn telomere length. We set quantiles in this model equal to those for the full sample to make the effect estimates between the two models more comparable. Fourth, we examined potential non-linearity of the exposure effects by altering the number of quantiles from 4 to 5, 10, and 15, and entering exposure variables as quadratic terms [42, 43]. Finally, to assess the sensitivity of our results to the use of NDVI as our measure of neighborhood greenness, we re-ran our primary analysis with two alternative measures: 1) the area of green space within 300 m of parental residential address (in square meters) based on the 2016 National Land Cover Database; [49] and 2) the distance to the nearest open access park (in meters) derived using a park shapefile from the Green Info Network [50]. We classified green as NLCD developed open space that is 80% vegetation, deciduous forest, evergreen forest, mixed forest, dwarf shrub, shrub, grassland, sedge, lichens, moss, pasture/hay, cultivated crops, woody wetlands, and emergent herbaceous wetlands. Confidence intervals have not been corrected for multiple comparisons, which should be taken into consideration when interpreting our findings given the number of tests performed. All analyses were conducted in R Version 4.1.2

## Results

Our sample consisted of 354 birth parents, 488 newborns, and 222 parental-infant pairs. Most birth parents had a college and/or graduate degree and identified as White or Latina (Table 1). Roughly 40% of parents were born outside of the U.S. As expected, telomere length was longer among newborn (mean T/S ratio = 1.5) than parental samples (mean T/S ratio = 1.0). Newborn and parental telomere length were weakly correlated with each other (ρ = 0.20) and inversely correlated with gestational age (ρ = −0.11) and parental age (ρ = −0.16), respectively (Supplementary Table S1). Built environment variables were moderately correlated with each other in the expected direction (ρ between −0.60 and 0.36, Supplementary Table S1). Poorer perceived neighborhood quality was weakly correlated with traffic (ρ = 0.14) and noise (ρ = 0.17), and NDVI (ρ = −0.16) in the expected direction (Supplementary Table S1). Perceptions of neighborhood quality, stress and depression were also weakly correlated with each other in the expected direction (ρ between 0.10 and 0.28, Supplementary Table S1).

Birth parents without a high school degree and those with a graduate degree lived in areas with less greenness and more noise, and reported poorer neighborhood quality, more perceived stress, and symptoms of depression, on average (Table 2). Asian and White participants lived in areas with the least noise and reported the lowest perceived stress and depression, on average, while Latina and Black participants lived in areas with the lowest average greenness and most noise and reported the worst neighborhood quality and highest amount of stress on average (Table 2). In contrast, Black participants lived in neighborhoods with markedly lower traffic on average than other racial and ethnic groups. Compared to immigrants, U.S born parents lived in neighborhoods with less greenness, traffic and noise, and reported better perceived neighborhood quality and less depression on average (Table 2). Telomere length of birth parents and newborns did not vary significantly with respect to nativity, race and ethnicity, or education (Table 2).

**Table 2:** Median level of built environment, psychosocial stressor and telomere length (T/S ratio) by nativity, race and ethnicity, and education (*N* = 620)^a^.

	NDVI	Traffic^b^	Noise^c^	Neighbor- hood quality^d^	Perceived stress	Depression	Gestational age (weeks)	Newborn T/S	Parental T/S
Immigrant									
Yes (*N* = 263)	0.29	505,994	48.8	40.0	5.0	7.0	39.0	1.48	1.02
No (*N* = 327)	0.25	346,172	46.0	38.0	5.0	6.0	39.0	1.48	1.04
Missing (*N* = 30)	0.25	292,695	48.6	36.5	6.0	6.0	39.0	1.51	1.03
*P*-value	<0.001	<0.001	<0.001	0.16	0.13	0.007	0.28	0.96	0.23
Race and Ethnicity									
Asian (*N* = 109)	0.29	428,059	46.7	39.0	5.0	6.0	39.0	1.47	1.05
Black (*N* = 24)	0.28	182,854	49.5	44.0	6.5	7.0	39.5	1.48	1.08
Latina (*N* = 208)	0.23	471,762	49.1	43.0	6.0	7.0	39.0	1.51	1.01
Other/Multi-Racial^e^ (*N* = 27)	0.30	486,692	47.4	35.0	5.0	7.5	39.0	1.47	1.06
White (*N* = 244)	0.29	411,692	45.9	37.0	4.0	5.0	39.0	1.48	1.03
Missing (*N* = 8)	0.22	603,835	50.2	38.0	6.0	10.0	39.0	1.63	1.19
*P*-value	<0.001	0.06	<0.001	<0.001	<0.001	<0.001	<0.001	0.64	0.55
Education									
<High school (HS) (*N *= 62)	0.24	392,040	49.5	41.5	7.0	8.0	39.0	1.54	0.96
HS or some college (*N* = 135)	0.27	433,369	46.7	37.0	5.0	6.0	39.0	1.48	1.04
College degree (*N* = 163)	0.29	413,791	45.8	37.0	4.0	5.0	39.0	1.46	1.02
Graduate degree (*N* = 236)	0.23	523,135	49.8	44.0	6.0	8.0	39.0	1.49	1.07
Missing (*N* = 24)	0.25	447,759	46.8	40.0	6.0	7.0	39.0	1.45	1.01
*P*-value	<0.001	0.06	<0.001	<0.001	<0.001	<0.001	<0.001	0.11	0.30

*P*-values are from the Kruskal–Wallis rank sum test of the null hypothesis that all groups are equal.

^a^Sample includes all participants with newborn telomere length (*N* = 266), parent telomere length (*N* = 132), or both (*N* = 222).

^b^Estimated number of motorized vehicles that passed through, started, or stopped within a 200 m circular buffer of participant’s residential address per month.

^c^24-h equivalent A-weighted decibel levels at 30 × 30 m resolution.

^d^Higher values indicate poorer perceived neighborhood quality.

^e^Includes Pacific Islanders, Native Americans, and other groups including multi-racial participants.

**Fig. 1:**
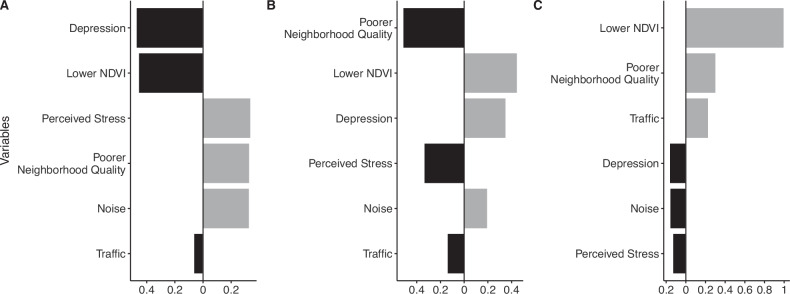
Weights representing the proportion of the positive and negative effects in the overall mixture in relation telomere length. Panel **A** shows weights for parental (*N* = 256), **B** for newborn (all, *N* = 385) and **C** for paired newborn (*N* = 175) samples. Note that the magnitude of positive weights can only be compared to other positive weights (not to negative weights) and vice versa. Black bars indicate negative weights, while gray bars indicate positive weights.

Quantile g-computation suggested a one quartile increase in the full mixture of adverse built environment variables and psychosocial stressors was not strongly associated with parental or newborn telomere length in the full study population (β = 0.01 [−0.03, 0.05] and β = −0.03 [95% CI −0.08, 0.01], respectively, Table 3). Among parental samples, poor neighborhood quality, noise and perceived stress contributed positively to the overall mixture effect, with weights indicating an association with longer telomere length, while depression, lower NDVI and traffic contributed to the overall negative effect (Fig. 1A). In contrast, among newborn samples, lower NDVI, depression and noise contributed positively to the overall mixture effect, while poor perceived neighborhood quality, perceived stress, and traffic contributed negatively (Fig. 1B).

**Table 3:** Quantile g-computation estimates and 95% confidence intervals for the mean difference in newborn and parental telomere length (T/S ratio) for a one quartile increase in the overall mixture of adverse built environment and psychosocial stressors.

	Parental T/S		Newborn T/S	
	*N*^a^	β (95% CI)	*N*^a^	β (95% CI)
All samples	256	0.01 (−0.03, 0.05)	385	−0.03 (−0.08, 0.01)
Paired samples	175	0.00 (−0.06, 0.05)	175	−0.08 (−0.15, −0.01)

Models control for parental age, race and ethnicity, education, pre-pregnancy BMI, and gestational age (newborn T/S only). When restricting to paired samples, models of newborn T/S additionally control for parental T/S.

^a^Sample sizes are lower than in Table 2 because the sample was restricted to participants with complete exposure data for built environmental and psychosocial stressor variables.

Restricting to parental-newborn paired samples and controlling for parental telomere length strengthened the association between the overall mixture and newborn telomere length. A one-quartile increase in the full mixture of adverse built environment and psychosocial stress measures was associated with a reduction in T/S ratio (β = −0.08 [−0.15, −0.01], Table 3), with traffic, noise and depression contributing negative weights (Fig. 1C).

In the interaction analysis, we did not observe associations between the overall mixture of environmental and social stressors with parental telomere length (Table 4). A one quartile increase in the adverse built environment and psychosocial stressor mixture was more strongly associated with a decrease in newborn telomere length among immigrant (β = −0.08 [−0.16, 0.00]) than U.S. born participants (β = −0.02 [−0.07, 0.04]) (*P*-value for interaction = 0.19) and among college educated participants (β = −0.07 [−0.12, −0.02]) but not those without a college degree (β = 0.03 [−0.07, 0.12]) (*P*-value for interaction = 0.08) (Table 4). The mixture was also more strongly associated with a decrease in newborn telomere length among White parents than parents from minoritized racial or ethnic groups (*P*-value for interaction = 0.33) (Table 4). Traffic contributed negatively to the overall association with newborn telomere length among the immigrant and college educated sample, consistent with our hypothesis, while lack of greenness contributed positively to the association, inconsistent with our hypothesis (Supplementary Figs. S2C, S3D). There was little consistency in terms of weights for other variables in our interaction analysis of newborn telomere length (Supplementary Figs. S2, S3).

**Table 4:** Quantile g-computation results of effect modification by nativity, race and ethnicity and education.

	Parental T/S			Newborn T/S		
	*N*	β (95% CI)	*P*-value	*N*	β (95% CI)	*P*-value
Nativity						
Immigrant	103	−0.02 (−0.09, 0.05)	0.37	152	–0.08 (−0.16, 0.00)	0.19
U.S. born	145	0.02 (−0.04, 0.07)		229	−0.02 (−0.07, 0.04)	
Race and ethnicity						
Minoritized	128	0.00 (−0.07, 0.07)	0.90	176	−0.02 (−0.09, 0.05)	0.33
White	106	0.01 (–0.05, 0.07)		173	−0.07 (−0.14, 0.00)	
Educational attainment						
No college degree	76	0.05 (–0.04, 0.14)	0.34	90	0.03 (−0.07, 0.12)	0.08
College degree	174	0.00 (−0.05, 0.05)		292	–0.07 (−0.12, −0.02)	

Quantile g-computation estimates and 95% confidence intervals for the mean difference in parental and newborn telomere length (T/S ratio) for a one quartile increase in the overall mixture of adverse built environment and psychosocial stressors. Models control for parental age, educational attainment (nativity and race and ethnicity models only), race and ethnicity (nativity and education models only), pre-pregnancy BMI, and gestational age (newborn T/S only). *P*-values are for interaction.

When we further stratified by race or ethnicity and educational attainment, we found that being an immigrant was associated with a decrease in parental telomere length per quartile increase in our mixture among ethno-racially-minoritized groups (β = –0.04 [−0.11, 0.04]) vs. β = 0.06 [−0.01, 0.13] for U.S. born, *P*-value for interaction = 0.06) and participants without a college degree (β = −0.05 [−0.17, 0.08]) for immigrants vs. β = 0.12 [−0.03, 0.28] for U.S. born, *P*-value for interaction = 0.10) (Supplementary Table S2). We found our mixture to be more strongly associated with a decrease in newborn telomere length among ethno-racially minoritized immigrants (β = −0.06 [−0.15, 0.02]) then U.S. born participants from ethno-racially minoritized groups (β = 0.02 [−0.06, 0.10]) (*P*-value for interaction = 0.14) and college-educated immigrants (β = −0.11 [−0.20, −0.02]) compared to college-educated U.S. born participants (β = −0.03 [−0.09, 0.03]) (*P*-value for interaction = 0.15) (Supplementary Table S2). Being an immigrant was also associated with stronger reductions in newborn-telomere length among White and non-college educated participants, but P-values for interaction were larger in these comparisons (0.48, and 0.19, respectively).

In sensitivity analysis of the main model, effect estimates for newborn telomere length changed minimally when controlling for infant sex or removing one outlier (Supplementary Table S3). Effect estimates for parental and newborn T/S became smaller with each increase in quantiles from 4 to 5, 10 and 15 quartiles, but the direction of effect estimate did not change (Supplementary Table S3). The sensitivity analysis including quadratic terms for exposure variables similarly showed no evidence of non-linear effects (Supplementary Table S3). In our evaluation of alternative measures of greenness, we found that replacing NDVI with the amount of greenspace or distance to the nearest park resulted in very similar overall effect estimates, with the direction of weights remaining consistent (negative weights for parental samples, positive weights for newborns) (Supplementary Table S4; Supplementary Figure S4).

## Discussion

Results from this study of a diverse pregnancy cohort from San Francisco suggest the combination of adverse built environment and psychosocial stressors was associated with a decrease in newborn telomere length, especially among paired samples for which we were able to control for parental telomere length (which is important given the relatively high heritability of telomere length) [51]. We also observed modestly stronger effects among immigrants and college-educated parents compared to parents born in the U.S. and with lower levels of educational attainment, although the evidence for interaction was only statistically significant at alpha = 0.20 and 0.10, respectively. Among paired samples, we observed a mean reduction in newborn T/S ratio among of −0.08 [−0.15, −0.01] per one quartile increase in the mixture of adverse built environment conditions and psychosocial stressors. This equates to roughly eight additional years of aging, based on an estimated telomere shortening rate of 0.01 T/S per year from a systematic review of studies in adults [52]. Our effect estimate is also stronger than the pooled estimates from recent meta-analyses of the relationship between newborn telomere length and parental stress (per one unit increase in parental psychosocial stress score) or air pollution (per 10 μg/m^3^ increase in in-utero exposure to fine particulate matter or nitrogen dioxide air pollution) [6, 53].

When looking at variable weights, only traffic consistently contributed to the negative association with newborn telomere length across paired samples (Fig. 1C) and the two subsets of the study population we considered (immigrants and college-educated, Supplementary Figs. S2C, S3D). Noise and depression additionally contributed negatively to the association among paired samples, noise and poor perceived neighborhood quality among immigrants, and perceived stress and depression among college educated parents, suggesting the factors driving the association with shorter telomeres may vary across populations. Exposure to greenspace has been associated with longer telomere length and lower telomere attrition in young children [18, 54]; however we found no evidence that greenness was associated with longer telomere length at birth.

The exposures we considered did not appear to influence telomere length among pregnant adults in our study. Our analysis was limited by the fact that we had only one measure of parental telomere length and were unable to look at attrition over time. It is possible that the exposures we considered influence the rate of telomere shortening, which we were unable to assess due to a lack of historical measures of telomere length. We also lacked residential histories to construct cumulative measures of exposure to neighborhood characteristics across the life course for participants that moved during their lifetimes.

We observed differences in neighborhood built environment characteristics for US born versus immigrant participants and across race and ethnicity and educational attainment. Most, but not all patterns we observed were consistent with the environmental justice literature documenting ethno-racially minoritized populations and lower socioeconomic status have less access to environmental amenities and higher exposure to environmental hazards [55–57]. Interestingly, participants with a graduate degree resided in areas with the least desirable built environment characteristics (less greenness, more traffic, and more noise, on average), while Black participants lived in areas with less traffic on average. This likely reflects recent gentrification and displacement of ethno-racially minoritized populations and populations with lower socioeconomic status from San Francisco’s urban core, which has been especially pronounced within the city’s Black population, and the suburbanization of poverty regionally [58, 59].

The level of greenness, traffic, and noise were correlated with individual participants’ perceptions of their neighborhood quality, and poorer neighborhood quality was in turn correlated with higher perceived stress and symptoms of depression during pregnancy. This is consistent with prior work suggesting that the neighborhood built environment can ‘get under the skin’ to influence mental health [60, 61]. For example, residential tree canopy has been associated with lower perceived stress among pregnant people with a history of anxiety or depression [62], while noise has been associated with depression during and after pregnancy in prior studies [14, 15].

Strengths of our study include its relatively large sample and racial/ethnic and socioeconomic diversity compared to prior studies of telomere length among pregnancy cohorts. Unlike other mixture approaches such as weighted quantile sum (WQS), our use of quantile g-computation allowed us to consider the joint effect of multiple exposures without assuming homogeneity in the direction of the relationships between individual exposures and our outcomes. Indeed, assessing single measures of environmental and social stressors, or summing across them, may not accurately capture how these kinds of exposures are likely to co-occur in complex ways that may have a joint or cumulative effects on health outcomes. Study limitations include that parental exposures and telomere length were measured at the same time, and the lack of time-varying measures of our outcomes or exposures of interest. Additional potential limitations include the use of cord blood to assess newborn telomere length, which can be susceptible to contamination from parental blood [63]. The remotely sensed NDVI measure we utilized also cannot distinguish between types of vegetation (i.e. tree canopy, grass, etc.), or public accessibility to green spaces that may have varying capacity to benefit health [64, 65]. We also lacked measures of psychological resilience, measured as an individual’s capacity to maintain positivity and satisfying social relationships amid stress or stressful experiences, which has been shown in one prior study to ameliorate the effects of parental stress on newborn telomere length [66]. Finally, while we did adjust for known covariates, as with all observational studies, we cannot rule out the possibility of individual or area-level residual confounding. While the diversity of our sample increases generalizability, further research is needed to confirm our findings in other populations.

To our knowledge, our study is the first to examine how mixtures of neighborhood built environmental factors and individual-level perceptions of psychosocial stressor exposures affect telomere length. We found that the mixture of neighborhood lack of greenness, traffic, and noise, coupled with individual-level poor perceptions of neighborhood quality, perceived stress, and depression is associated with decreased telomere length among newborns, with slightly stronger effects among immigrants and college-educated birth parents. Future studies should extend the geographic scope of study populations to assess the extent to which effects might vary regionally and across demographic groups.

## Supplementary information

Supplementary Information

## Data Availability

Per University of California, San Francisco Institutional Review Board approval, the data that support the findings of this study are restricted for transmission to those outside the primary investigative team. Data sharing with investigators outside the team requires IRB approval. Requests may be submitted to the Program on Reproductive Health and the Environment (PRHE).
