# Supplementary material for: Neighborhood built environment, psychosocial stressors, and telomere length of birth parents and their newborns from San Francisco, California
Source: J Expo Sci Environ Epidemiol. Author manuscript; Available in PMC 2026 Mar 6. (PMC12960206; doi:10.1038/s41370-025-00797-9)
Supplement: Suplementary Information [file NIHMS2151260-supplement-Suplementary_Information.docx]

**Supplementary Information**

Neighborhood built environment, psychosocial stressors, and telomere length of birth parents and their newborns from San Francisco, California

Contents

[Contents 1](#_Toc189044058)

[Table S1. Spearman’s correlation coefficients between telomere length (T/S ratio), built environment and psychosocial stressors (N=620 samples with maternal or newborn T/S) 2](#_Toc189044059)

[Table S2. Quantile g-computation results of effect modification by nativity, stratified by race and ethnicity and educational attainment. 3](#_Toc189044060)

[Table S3. Quantile g-computation estimates and 95% confidence intervals from sensitivity analyses 4](#_Toc189044061)

[Table S4. Quantile g-computation estimates and 95% confidence intervals using alternative green space measures 5](#_Toc189044062)

[Figure S1. Flow diagram of study population creation 6](#_Toc189044063)

[Figure S2. Weights from g-computation models of effect measure modification by nativity. 7](#_Toc189044064)

[Figure S3. Weights from g-computation models of effect measure modification by educational attainment. 8](#_Toc189044065)

[Figure S4. Weights from g-computation models using alternative greenness measures. 9](#_Toc189044066)

Table S1. Spearman’s correlation coefficients between telomere length (T/S ratio), built environment and psychosocial stressors (N=620 samples with maternal or newborn T/S). Values in bold are statistically significant at alpha = 0.05.

|  | Traffic | Noise | Neighborhood quality | Perceived stress | Depression | Age | BMI | Gestational age | Parental T/S | Newborn T/S |
| --- | --- | --- | --- | --- | --- | --- | --- | --- | --- | --- |
| NDVI | **-0.60** | **-0.30** | **-0.16** | -0.03 | -0.09 | 0.05 | **-0.11** | 0.04 | 0.08 | -0.08 |
| Traffic | 1 | **0.36** | **0.14** | -0.02 | 0.01 | -0.03 | 0.06 | -0.01 | -0.05 | 0.02 |
| Noise |  | 1 | **0.17** | **0.10** | **0.13** | -0.03 | **0.11** | -0.01 | 0.03 | 0.03 |
| Neighborhood quality |  |  | 1 | **0.24** | **0.28** | **-0.14** | **0.16** | -0.03 | 0.01 | -0.10 |
| Perceived stress |  |  |  | 1 | **0.56** | **-0.13** | **0.16** | -0.07 | 0.05 | -0.08 |
| Depression |  |  |  |  | 1 | -0.03 | **0.24** | -0.05 | 0.01 | -0.06 |
| Age |  |  |  |  |  | 1 | -0.02 | 0.08 | **-0.16** | -0.02 |
| BMI |  |  |  |  |  |  | 1 | **-0.11** | -0.04 | 0.03 |
| Gestational age |  |  |  |  |  |  |  | 1 | -0.01 | **-0.11** |
| Parental T/S |  |  |  |  |  |  |  |  | 1 | **0.20** |

Table S2. Quantile g-computation results of effect modification by nativity, stratified by race and ethnicity and educational attainment. Quantile g-computation estimates and 95% confidence intervals for the mean difference in parental and newborn telomere length (T/S ratio) for a one quartile increase in the overall mixture of adverse built environment and psychosocial stressors. Models control for parental age, educational attainment (race and ethnicity model only), race and ethnicity (education model only), pre-pregnancy BMI, and gestational age (newborn T/S only). P-values are for interaction.

|  | **Parental T/S** | | | **Newborn T/S** | | |
| --- | --- | --- | --- | --- | --- | --- |
|  | **N** | **β (95% CI)** | **P-value** | **N** | **β (95% CI)** | **P-value** |
| Nativity x Race and ethnicity | |  |  |  |  |  |
| Racially minoritized immigrants | 83 | -0.04 (-0.11, 0.04) | 0.06 | 119 | -0.06 (-0.15, 0.02) | 0.14 |
| Racially minoritized U.S. born | 57 | 0.06 (-0.01, 0.13) |  | 86 | 0.02 (-0.06, 0.10) |  |
| White immigrants | 17 | 0.03 (-0.13, 0.20) | 0.92 | 30 | -0.10 (-0.27, 0.08) | 0.48 |
| White U.S born | 87 | 0.02 (-0.05, 0.10) |  | 142 | -0.03 (-0.10, 0.05) |  |
| Nativity x Education | |  |  |  |  |  |
| College educated immigrants | 56 | 0.00 (-0.08, 0.07) | 0.63 | 94 | -0.11 (-0.20, -0.02) | 0.15 |
| College educated U.S. born | 115 | 0.02 (-0.04, 0.08) |  | 196 | -0.03 (-0.09, 0.03) |  |
| Non college educated immigrants | 47 | -0.05 (-0.17, 0.08) | 0.10 | 58 | -0.03 (-0.19, 0.11) | 0.19 |
| Non college educated U.S. born | 29 | 0.12 (-0.03, 0.28) |  | 32 | 0.14 (-0.07, 0.36) |  |

Table S3. Quantile g-computation estimates and 95% confidence intervals from sensitivity analyses**.** Models control for parental age, race/ethnicity, education, pre-pregnancy BMI, and gestational age (newborn T/S only).

|  | **Parental T/S** | | **Newborn T/S** | |
| --- | --- | --- | --- | --- |
|  | **N** | **β (95% CI)** | **N** | **β (95% CI)** |
| Control for infant sex | -- | -- | 385 | -0.03 (-0.08, 0.01) |
| Outlier removed | -- | -- | 384 | -0.04 (-0.08, 0.01) |
| 5 quantiles | 256 | 0.02 (-0.02, 0.05) | 385 | -0.02 (-0.05, 0.02) |
| 10 quantiles | 256 | 0.01 (-0.01. 0.03) | 385 | -0.01 (-0.03, 0.01) |
| 15 quantiles | 256 | 0.01 (-0.01, 0.02) | 385 | -0.01 (-0.02 0.01) |
| Quadratic terms | 256 | 0.01 (-0.03, 0.05) | 385 | -0.04 (-0.08, 0.01) |

Table S4. Quantile g-computation estimates and 95% confidence intervals using alternative green space measures**.** Models control for parental age, race/ethnicity, education, pre-pregnancy BMI, and gestational age (newborn T/S only).

|  | **Parental T/S** | | **Newborn T/S** | | **Paired Newborn T/S** | |
| --- | --- | --- | --- | --- | --- | --- |
|  | **N** | **β (95% CI)** | **N** | **β (95% CI)** | **N** | **β (95% CI)** |
| Green area within 300m (NLCD) | 257 | 0.02 (-0.02, 0.06) | 387 | -0.02 (-0.07, 0.03) | 176 | -0.09 (-0.17, -0.02) |
| Distance to nearest open access park | 258 | 0.01 (-0.04, 0.05) | 388 | -0.03 (-0.08, 0.02) | 177 | -0.09 (-0.16, -0.02) |

# **Figure S1. Flow diagram of study population creation**


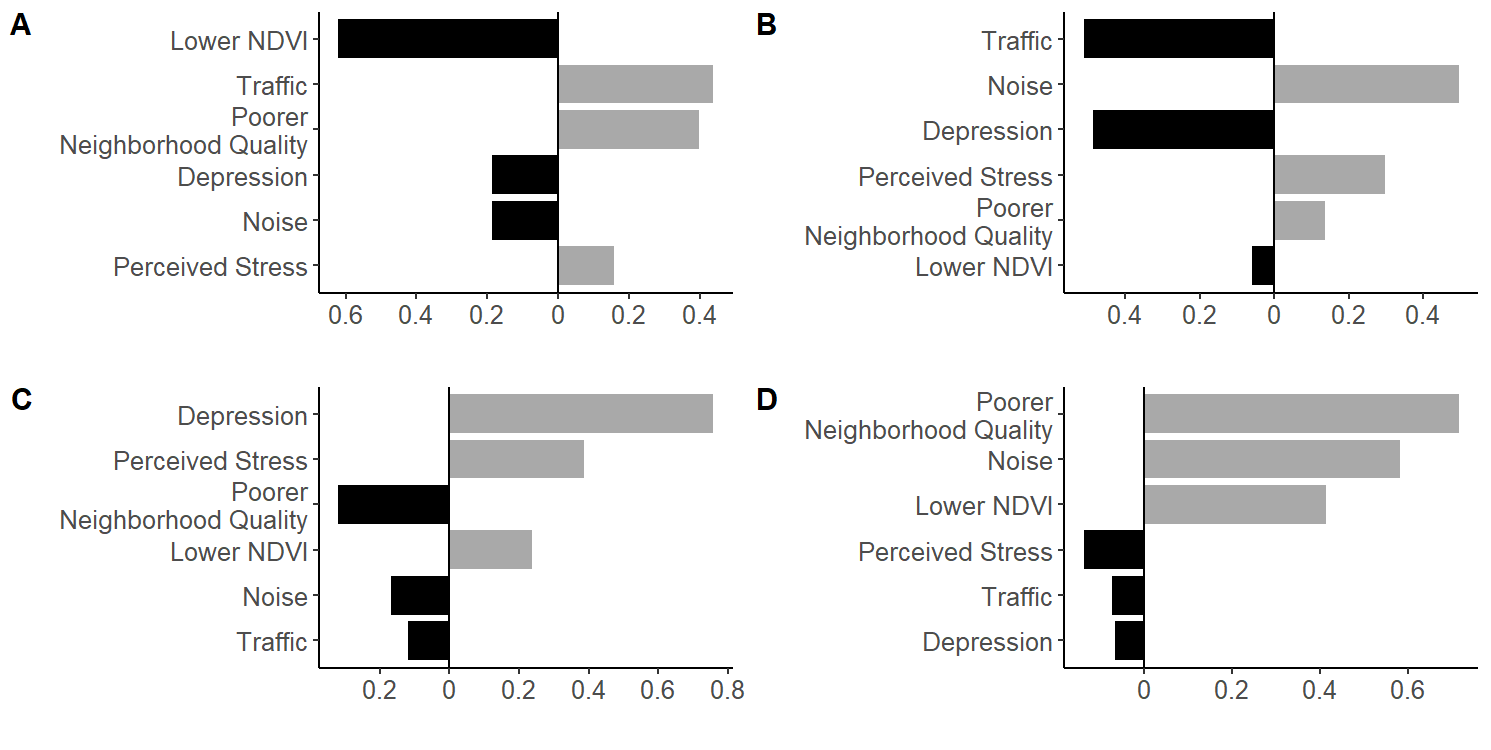


Figure S2. Weights from g-computation models of effect measure modification by nativity. Weights representing the proportion of the positive and negative effects in the overall mixture in relation to parental telomere length among A) immigrants (N=103) and B) U.S born (N=145) participants, and newborn telomere length among C) immigrant parents (N=152) and D) U.S. born parents (N=229). Note that the magnitude of positive weights can only be compared to other positive weights (not to negative weights) and vice versa. Black bars indicate negative weights, while gray bars indicate positive weights.


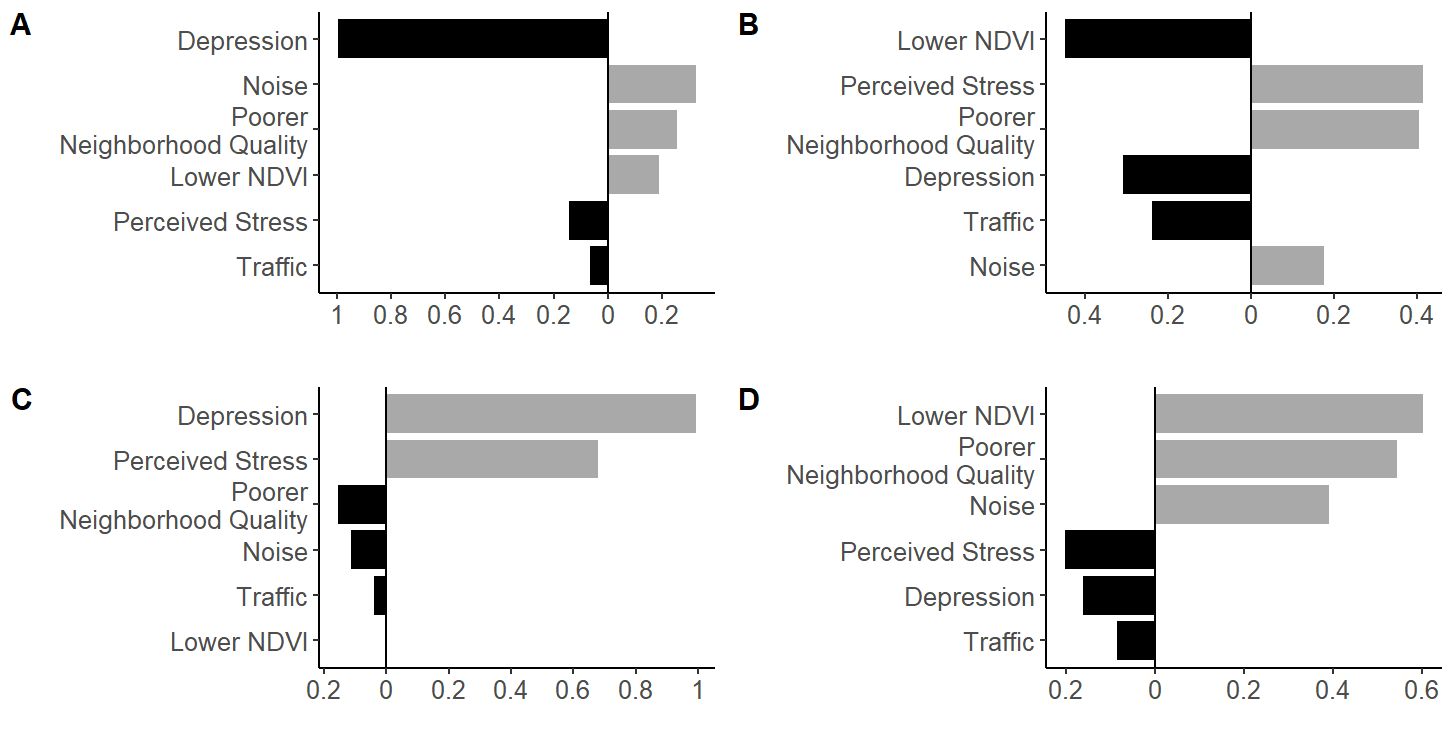


Figure S3. Weights from g-computation models of effect measure modification by educational attainment. Weights representing the proportion of the positive and negative effects in the overall mixture in relation to parental telomere length for participants+ that A) have less than a college degree (N =76) and B) a college degree (N =174) and newborn telomere length for newborns with parents that C) have less than a college degree (N=90) and D) a college degree (N=292). Note that the magnitude of positive weights can only be compared to other positive weights (not to negative weights) and vice versa. Black bars indicate negative weights, while gray bars indicate positive weights.


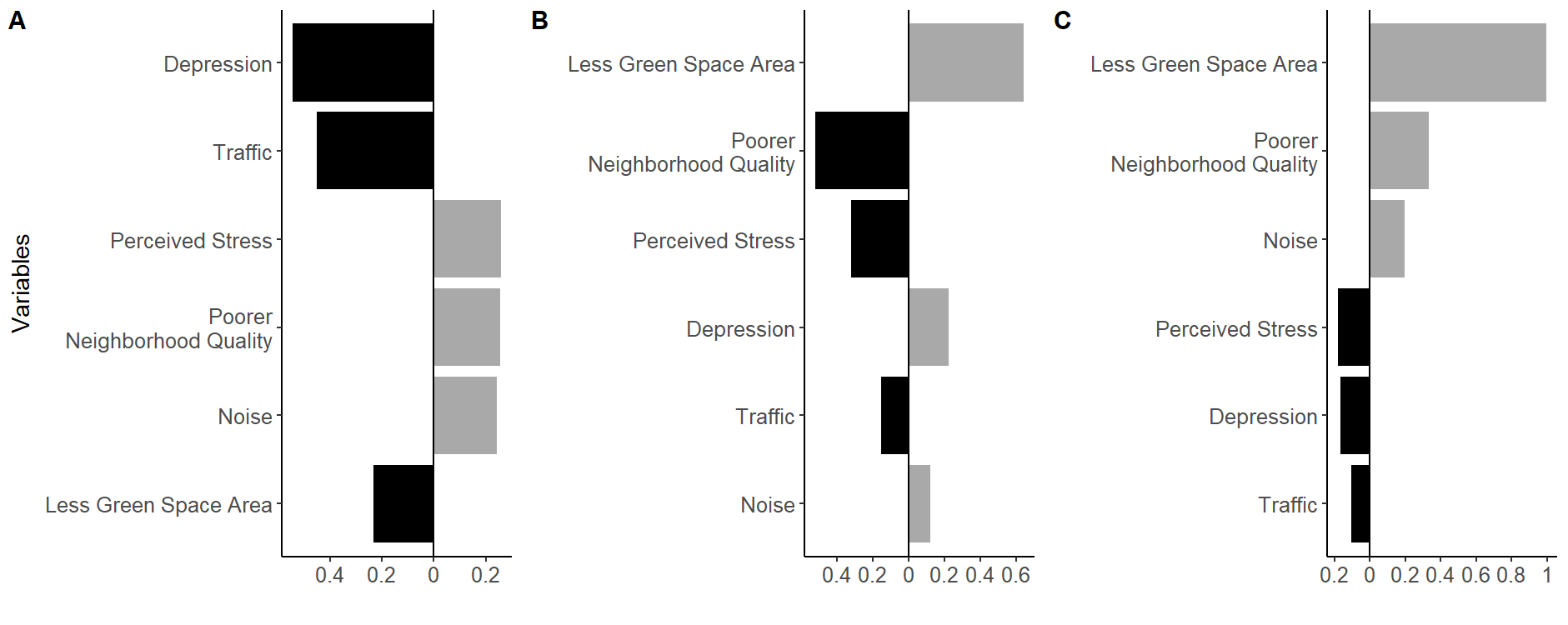


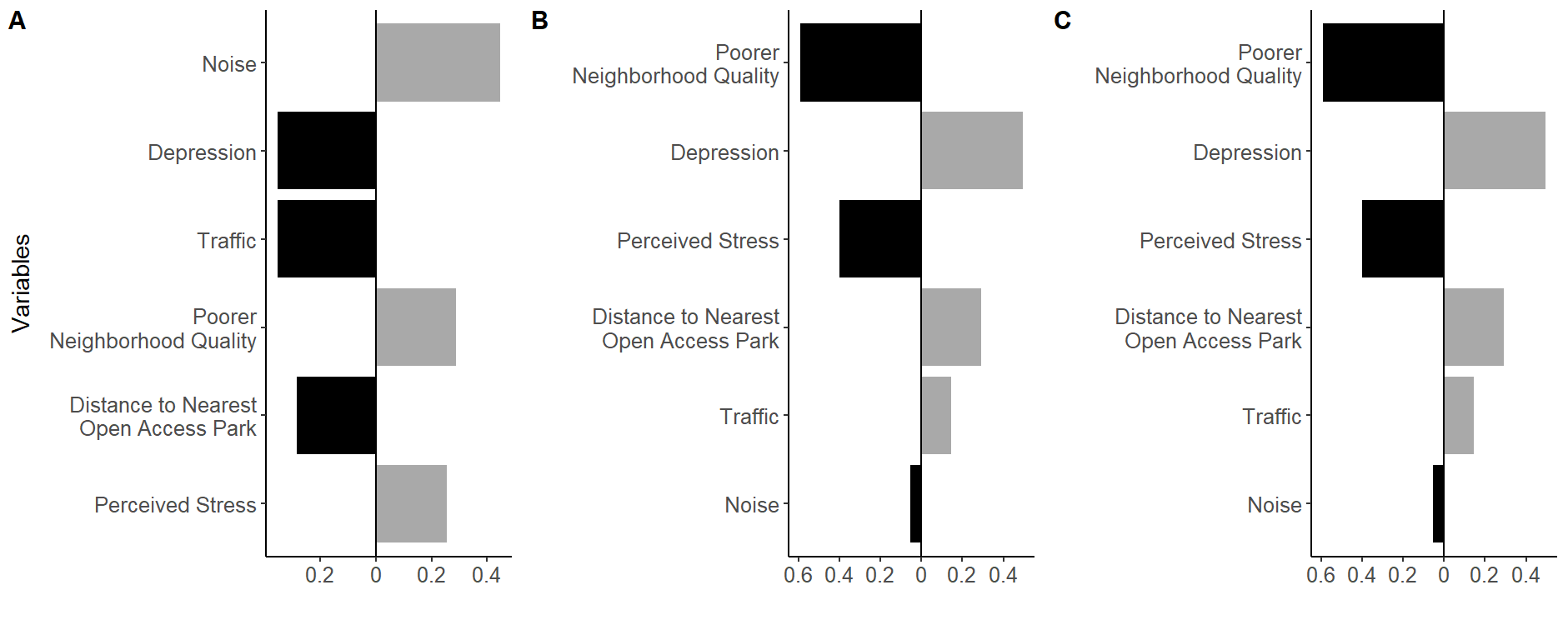


Figure S4. Weights from g-computation models using alternative greenness measures. Weights representing the proportion of the positive and negative effects in the overall mixture in relation to A) parental, B) newborn, and C) paired newborn T/S ratio using from green area within 300m (top) and distance to nearest open access park (bottom) as the greenness measure. Note that the magnitude of positive weights can only be compared to other positive weights (not to negative weights) and vice versa. Black bars indicate negative weights, while gray bars indicate positive weights.
